# Supplementary material for: Are carbon emissions trading and green financial instruments synergistic? -Comprehensive quantitative research based on content analysis
Source: PLoS One. 2024 Mar 7;19(3):e0298601. doi: 10.1371/journal.pone.0298601 (PMC10919601; doi:10.1371/journal.pone.0298601)
Supplement: S1 Data — (ZIP) [file pone.0298601.s001.zip › Data/地级市Z_F工作报告.zip!/╒π╜¡/╔▄╨╦╩╨/└┤╫╘╣1⁄2╓┌║┼í╛┬φ┐╦╩2╛▌═°í┐_2015.docx]

各位代表：

　　现在，我代表绍兴市人民政府，向大会作工作报告，请予审议，并请市政协委员和其他列席人员提出意见。

　　一、2014年主要工作

　　2014年是全面深化改革的开局之年，是新一轮大城市建设的启动之年。一年来，面对复杂的外部环境、严峻的经济形势和繁重的改革任务，市政府认真贯彻中央和省各项方针政策，全面落实市委“重构绍兴产业、重建绍兴水城”战略部署，牢牢把握发展大势，扎实做好各项工作，保持了经济社会持续健康发展的良好态势。全市实现地区生产总值4266亿元，增长7.5%；公共财政预算收入317亿元，增长8.3%；城镇和农村常住居民人均可支配收入分别达到43167元和23539元，增长9.1%和10.5%。市七届人大四次会议确定的主要预期目标基本完成。

　　一年来，我们紧紧围绕“改革创新促发展、求真务实惠民生”工作基调，突出抓了以下八方面工作。

　　（一）着力深化各项改革。坚持改革统领，把改革创新贯穿于各领域各环节。积极推进行政审批制度改革，推广柯桥区高效审批省级试点经验，在滨海新城试行核准目录外企业投资项目不再审批，设立市级“中介超市”，完成审批事项“归零清理”，市级行政审批事项从843项减少到506项。大力推进“四张清单一张网”改革，编制并向社会公布政府权力清单和责任清单、企业投资项目负面清单、财政专项资金管理清单，建成政务服务网，市级部门行政权力从8781项减少到4045项。加快推进经济体制改革，实施市场监管体制调整和商事制度改革，探索企业综合评价分类管理制度。健全国资监管体系，组建震元健康产业集团、文化产业发展投资公司、广播电视传媒集团、环卫集团，开展绍兴银行人事制度改革，完善绍兴报业传媒集团法人治理结构。统筹推进城管、旅游等社会领域改革，推动社会力量办医、办学、办文化，基层卫生综合改革和公立医院改革列入国家重点联系市。深入推进农村综合改革，实施农村集体资产股份制改革，启动“三位一体”农民合作经济组织体系试点，开展农房确权发证，县级农村产权交易平台实现全覆盖。稳步推进政府运行机制改革，实行全口径财政预算管理，首次公开部门“三公”经费预算，有序推进市级机关公车改革。开展机关事业单位控编减编，清理规范编外用工。调整完善市与越城区、开发区体制机制，推动权力下放和人员下移。

　　（二）全力稳定经济运行。扩大有效投资，建立产业项目准入评估制度，制定项目市内流转办法，开展政府投资项目现场监督，完成固定资产投资2305亿元、增长15.1%，其中工业投资1106亿元、增长10.6%。高度重视招商引资，越商回归实到资金192亿元，实到外资6.7亿美元。建设外贸综合服务平台，开展“百场展会拓市场”活动，实现外贸出口298亿美元、增长6.6%。推进商品市场提升发展，建成金帝银泰城、诸暨国际商贸城、新昌世贸广场等一批商贸综合体，实现社会消费品零售总额1487亿元、增长12.8%。培育电子商务等新模式，实现网络零售额165亿元、增长65.5%。加强政银企联动，开展金融支持经济百日服务活动，新增社会融资1232亿元，其中直接融资384亿元。稳步推进城镇低效用地再开发，消化批而未供土地3万亩，盘活存量用地1万亩。培育壮大新兴市场主体，完成“个转企”4467家、“小升规”462家。切实防范经济金融风险，出台企业帮扶、兼并重组等系列政策，设立应急转贷资金，依法打击恶意逃废债，有力有效处置银行不良资产，平稳有序处置经营风险企业。加强经济交流和协作，开展与新加坡等合作，荣获国际友城交流合作奖。制订接轨上海、对接上海自贸区三年行动计划，全面落实援疆、援藏、援青和山海协作等各项任务。

　　（三）大力调整产业结构。实施“四换三名”工程，加大腾笼换鸟、机器换人、电商换市、空间换地力度，积极培育名企、名品、名家。完善战略性新兴产业扶持政策，培育发展高端装备制造、生命健康、文化旅游等产业，战略性新兴产业产值占工业总产值比重达到30.1%。加快传统产业技术改造步伐，推进印染等“三高一低”产业转型升级，淘汰1625家企业的落后产能，单位工业增加值能耗下降6.2%。促进工业化、信息化融合，柯桥区、诸暨市成为省“两化”深度融合示范区，上虞区、新昌县成为省绿色安全制造信息化示范区。加快建筑产业现代化步伐，实现建筑业总产值6178亿元、增长11.9%。扎实推进现代服务业“310工程”和“百项百亿”计划，完成服务业投资1183亿元、增长19.8%，服务业增加值占生产总值比重提高到43.6%。积极推进全市游，分步实施“绍兴人免费游绍兴”，实现旅游总收入652亿元、增长11.6%。加快推进农业现代化，建成省市级现代农业园区28个、粮食生产功能区11.7万亩，粮食生产实现11年连续增长。开展“八倍增、两提高”科技领航行动，新认定国家高新技术企业172家，预计研究与试验发展经费支出占生产总值比重达2.2%，国家可持续发展实验区建设通过验收。优化人才住房保障等政策，引进硕士及副高以上人才2560名。

　　（四）合力推进城乡统筹。编制实施绍兴市城市发展战略纲要，完成大城市发展战略11个专题研究。谋划推进“一环六横八纵”中心城市快速路网建设，杭长高铁绍兴段如期建成，31省道北延越城区段基本建成，104国道柯桥区高架、绍诸高速诸暨延伸线开工建设，杭绍台高速前期工作高效推进，越城区至上虞区公交开通运营。积极推进绍兴高新区迪荡湖整治建设，加快袍江“两湖”区域开发，进一步完善滨海新城基础设施，建成开放科技中心、文化中心、奥体中心。制定绍兴智慧城市概念规划，完善通信等信息基础设施。系统治理城市交通拥堵，完善机动车、非机动车、公交和水运“四网”规划，新增公共自行车1万辆。

顺利推进越城区人力客运三轮车整治，取消东街夜市。完善城中村拆迁安置办法，基本完成越城区小区移交工作。积极建设诸暨城东新城。推进嵊新区域集约发展。健全小城市分类培育机制。深入开展美丽乡村示范区建设，改造农房1.1万户，嵊州市成为省级美丽乡村先进县。

　　（五）强力治理生态环境。编制绍兴水城概念性规划，积极构建现代水城。大力推进“五水共治”，组织实施“双百双千”工程，完成投资595亿元，钦寸水库、永宁水库顺利截流，鉴湖二期、梅龙湖等项目完工，“八湖”植物景观工程加快建设。推行分级“河长制”，整治黑臭河199条、238公里，基本消灭垃圾河，五类、劣五类水质断面由14个减少到8个。开展农村生活污水治理，完成治理村527个，新增受益农户20万户。实施污水分质提标和印染废水集中预处理。加强防洪、排涝、城市供水等水利基础设施建设。稳步推进“五气合治”，实施大气污染防治三年行动计划，淘汰黄标车及老旧车3.4万辆，淘汰燃煤锅炉878台，市区空气质量优良率达到67.4%，PM2.5国控点监测指数下降11.3%。建立工业污泥、建筑泥浆、工程渣土“五统一”制度，实现市场化运作、资源化利用。全面推进“三改一拆”，开展“无违建镇村”创建，拆除违法建筑1506万平方米，改造旧住宅区、旧厂区和城中村882万平方米。扎实推进“四边三化”，规范整治高速公路沿线广告牌。积极推进“森林绍兴”、“花卉绍兴”建设，选定香榧为绍兴市树，省级森林城市实现区、县（市）“满堂红”。新昌县成为国家级生态县，全市生态环境公众满意度比上年提高4.6个百分点。

　　（六）尽力改善群众生活。全市公共财政用于民生支出260亿元、增长14%，占公共财政预算总支出的74.9%，市七届人大四次会议确定的十方面民生实事如期完成。持续增加城乡居民收入，及时落实上级有关政策，企业退休人员基本养老金人均每月增加238元，城乡居民基础养老金每月增加25元，最低月工资标准提高160元。完善就业创业促进体系，新增城镇就业11万人，帮助城镇失业人员再就业4万人，城镇登记失业率为2.8%。健全社会保障体系，启动全民参保登记工作，实施城乡居民大病保险制度，实行职工医疗互助保障市级统筹，新建改建农村养老服务中心507家，开工建设保障性安居住房1.9万套、竣工7920套，再次荣获全国七星级慈善城市称号，成为全国适度普惠型儿童福利制度建设试点市。大力促进教育惠民、卫计惠民、文体惠民。规范外来务工人员子女入学，推行义务教育学校教师交流制度，义务教育标准化学校创建率全省第一，浙江树人大学落户柯桥区。建成全市统一的预约诊疗平台，启动分级诊疗试点，实现全省联网就医“一卡通”，落实“单独两孩”政策。开展“文化三下乡”活动，新建农村文化礼堂126家。组织“全民健身与省运同行”系列活动，成功承办第十五届省运会。

　　（七）努力促进社会和谐。全力争创全国文明城市，省评成绩居全省同类城市第一。深入推进社会治安防控体系建设，严厉打击各类违法犯罪活动。实施重大决策社会稳定风险评估，加大信访积案化解力度，信访总量下降8%。高度重视安全生产、食品药品安全等工作，安全生产事故起数、死亡人数分别下降2.7%和9.8%，乡镇食品药品监管网络实现全覆盖。推行家禽“杀白上市”，做好人感染H7N9禽流感等重大疫情防控工作。广泛开展“六五”普法。稳步发展老龄、妇女儿童、残疾人、民族宗教、对台事务、文史档案、红十字等事业，气象等防灾减灾体系建设居全省前列。支持国防和军队建设，扎实推进国防动员、人民防空、国家安全等工作，连续六次成为省“双拥模范城”。

　　（八）致力转变工作作风。认真开展政府系统党的群众路线教育实践活动，严格执行中央八项规定和省、市有关精神，聚焦“四风”问题，大力整治文风会风、酒局牌局、公款旅游，全市“三公”经费支出、因公出国（境）人次分别下降37%和68%。充分发挥审计、监察、政府督查等作用，深入开展领导干部社会化评价、机关干部“一对一”联系服务企业、行政主职暗访检查、民情通等工作，进一步提高机关效能。务实办理人大代表建议、政协提案，按时办结率达到100%。

　　各位代表，过去一年的成绩来之不易。这得益于省委、省政府和市委的正确领导，得益于全市人民的共同努力。在此，我谨代表市人民政府，向全市人民，向给予政府工作大力支持的人大代表、政协委员、各民主党派、社会各界人士，向所有关心、支持和参与绍兴发展的同志们、朋友们，致以崇高的敬意和衷心的感谢！

　　我们也清醒地看到，我市经济社会发展中还存在不少问题，政府工作还有很多不足。保持经济平稳较快增长面临挑战，2014年地区生产总值增幅低于预期0.5个百分点，部分企业生产经营比较困难，企业资金链断裂引发局部金融风险的隐患仍然存在；经济结构性问题比较突出，经济发展的质量和效益有待提高；城乡基础设施、公共服务还不平衡，大城市融合任务繁重，农民持续增收压力比较大；有效投资后劲不足，具有引领性、带动性的大项目好项目不够多；创新驱动能力不够强，高端人才比较缺乏；生态环境治理任重道远，治理工作的科学性、系统性、持续性有待加强；政府职能转变还要加快，机关作风建设有待深化，一些部门和地方形式主义、官僚主义、享乐主义、奢靡之风依然存在，一些政府工作人员改革理念、法治意识、担当精神不强，一些领域腐败现象时有发生。对此，我们一定要保持清醒头脑，坚持求真务实，更好履职尽责，努力推动绍兴科学发展、文明发展、理性发展。

　　二、2015年目标任务和重点工作

　　2015年是全面深化改革的关键之年，是全面推进法治绍兴建设的开局之年，也是全面完成“十二五”规划的收官之年。做好今年工作，既有许多有利条件，也有不少实际困难。世界经济缓慢复苏，我国发展仍处于重要战略机遇期，宏观环境呈现许多新常态。我们要深刻认识新常态，准确把握我市正处于发展路径转折期、大城市建设关键期、转型升级攻坚期的阶段性特征，坚持为民取向，坚持问题导向，勇于改革、敢于担当，科学谋划、实干兴市，确保经济平稳健康发展和社会和谐稳定。

　　2015年工作的指导思想是：认真贯彻党的十八大和十八届三中、四中全会精神，以省“八八战略”为总纲，全面实施“重构绍兴产业、重建绍兴水城”战略部署，主动适应新常态，坚持稳中求进，坚持改革创新，坚持依法治市，着力提高经济发展质量和效益，着力加快大城市和现代水城建设，着力提升人民群众满意度，圆满完成“十二五”规划确定的各项目标任务，为干好“一三五”、实现“四翻番”奠定坚实基础。

　　建议2015年全市经济社会发展的主要预期目标是：地区生产总值增长7.5%以上；一般公共预算收入增长7.5%以上；城镇和农村常住居民人均可支配收入分别增长8%和9%以上。节能减排指标完成省下达任务。重点抓好以下五方面工作。

　　（一）坚持稳中求进，全面促进经济提质增效

　　扩大提升有效投资。优化投资结构，突出抓好重大产业项目、工业技术改造、重大基础设施、生态环境保护等方面的投资，力争固定资产投资增长12%以上。切实加大招商引资力度，创新以企引企、民外合璧等招商方式，紧盯央企、名企，承接上海等高端制造业转移，力争实到外资8亿美元、越商回归直接投资200亿元以上。把开发区作为招商引资、有效投资主战场，大力推进开发区强主业、再创业。科学谋划政府性投资，规范项目全过程管理，合理控制政府性债务。多方激活民间投资，发挥上市企业、龙头企业带动作用，鼓励民间资本进入社会事业领域、参与重大项目建设。下大力气改善投资服务环境，进一步营造安商、亲商、富商的良好氛围。

　　加快外贸转型发展。积极参与丝绸之路经济带和21世纪海上丝绸之路、长江经济带等国家战略的实施，主动对接上海自贸区。组织企业“抱团参展”，加大市场开拓力度，扩大服务贸易等出口，确保外贸出口增长7%以上。深入实施“品牌兴贸”战略，推动出口产业集群化、产品高端化。启动海关特殊监管区申报，完善外贸综合服务平台，优化外贸环境。支持先进技术、关键设备和紧缺资源进口，鼓励企业“走出去”并购优质资产、创建生产基地、构建营销网络。积极推进与新加坡经贸合作、与北京市西城区战略合作，加强与国际友城产业对接，继续做好对口支援、山海协作、援助新疆阿瓦提等工作。

　　优化现代产业体系。坚持工业强市、实业兴市，修订产业发展和提升规划，做强做大智能装备、生命健康、时尚等新兴产业，力争战略性新兴产业产值占工业总产值比重达到三分之一。全面推进“四换三名”，改造提升纺织、机械等传统产业，保护发展黄酒等历史经典产业，加快印染产业减量提质，坚决淘汰一批落后产能。推进工业化、信息化深度融合，大力发展信息经济，鼓励发展新技术、新产品、新业态、新商业模式，培育一批新的经济增长点。开展建筑产业、住宅产业现代化国家级“双试点”，加快建筑大市向建筑强市转变，促进房地产市场平稳健康发展。深化全国质量强市示范城市创建，建设国家级检测试验科研基地。坚持走现代农业发展道路，坚守粮食安全和耕地保护红线，加快农业科技创新和农技推广，引导茶叶、香榧等主导农产品扩大网上销售，加强农田水利设施建设，确保粮食播种面积218万亩以上，新建成省市级现代农业园区16个。继续实施现代服务业“310工程”，大力发展现代金融、现代物流、工业设计等现代服务业，培育文化、健康、养老、休闲等消费热点，力争社会消费品零售总额增长11%以上。整合全市旅游资源，积极发展古城旅游、水城旅游、文化休闲旅游，加快推进“中国兰亭”、镜湖旅游综合体、迎恩门风情水街、黄酒文化园、绍兴青旅羊山生态城、杭州湾商旅综合体等重点项目建设，深入推广“老绍兴、醉江南”旅游形象品牌。

　　务实做好企业服务。加大龙头骨干企业培育力度，鼓励企业兼并重组、做强做大。落实小微企业、成长型企业扶持举措，做好“个转企、小升规、规改股、股上市”工作。积极推动金融改革创新，做大做强地方法人银行，扩大上市融资、债权融资规模，鼓励企业运用信托、融资租赁等金融工具，重视做好银行不良资产处置工作。加大金融支持实体经济力度，提高房地产等抵押标准，争取社会融资1000亿元，维护良好的金融生态环境。深入开展城镇低效用地再开发，完善城镇土地使用税政策，盘活存量土地。编制涉企收费清单，严格清理涉企收费，规范管理中介机构，切实减轻企业负担。充分发挥行业协会作用，加强困难企业帮扶，综合治理恶意逃废债行为，防范和有效化解企业经营风险。

　　（二）坚持改革创新，全面增强发展动力活力

　　深化体制机制改革。积极争取、统筹抓好各项改革试点，形成一批可复制、可推广的改革经验。启动杭州都市经济圈转型升级综合改革试点，深化要素配置市场化改革，建立健全要素分层定价、企业分类指导、生态环境财政奖惩等制度。推动行政审批制度改革向纵深推进，完善企业投资项目高效审批服务机制，实行企业“零地”投资项目政府不再审批，开展部分项目不再环评试点。稳步推进政府职能转变和机构改革，建立“四张清单”动态调整机制，提升政务服务网功能。深化事业单位分类改革，规范收入分配管理。加快推进国资国企改革，充分发挥国有企业作用。抓好柯桥区省级农村改革试验区建设，深化嵊州市等乡镇治理模式创新，基本完成农村土地承包经营权、农民宅基地使用权、农房所有权确权登记发证，全面完成农村集体资产股份制改革、乡镇农村产权交易平台建设等改革任务。

　　实施创新驱动战略。深化国家创新型试点城市、国家知识产权示范城市创建，开展新昌县省级科技体制改革试点。紧扣“八倍增、两提高”目标，支持企业加大新产品、新技术、新应用投入力度，力争研究与试验发展经费支出占生产总值比重2.3%以上。培育壮大创新主体，重点培育科技领军企业10家、国家高新技术企业100家、省级科技型企业500家。鼓励企业自主创新，加强企业研究机构建设。推广“一个产业一个研究院”模式，加大重点产业技术攻关、科技成果转化力度，力争实现新产品产值3280亿元、增长15%。完善区域创新体系，深化对外科技合作，强化创新平台支撑，加快推进绍兴高新区科技城建设。

　　大力建设人才强市。把人才作为区域核心竞争力，健全人才培养、引进、服务体系，改善育才、引才、用才环境。深入实施“330海外英才计划”，继续开展“百名博士绍兴行”等活动，推进外国专家工作站建设，引进一批高层次专家、高技能人才、高素质创新团队。切实加强教师、医技、法律、科研、农技等专业队伍建设，支持企业加强技能人才培育。关心关爱企业经营者，实施企业家素质提升“百千万”工程，努力造就一支勇于进取、富有责任的越商队伍。

　　（三）坚持统筹发展，全面加快新型城市化步伐

　　突出抓好三区融合。全面落实绍兴市城市发展战略纲要，完善专项规划体系。加快现代水城建设，打造镜湖现代水城核心区，推进曹娥江两岸、“八湖”区域保护开发，努力促进水运复兴。统筹越城区、柯桥区、上虞区重大基础设施、重要公共设施建设，制定中心城市重大建设项目实施计划，有序推进路网、电网、水网、气网等建设改造，提高城市基础设施综合配套和衔接水平。加快构建“一环六横八纵”中心城市快速路网，联接三区交通体系。优化中心城市产业布局，促进传统产业互补、优势产业合作、新兴产业共建，实现错位发展。坚持“五个不变”原则，充分发挥各区共建大城市的积极性、创造性。稳妥制订有利于三区发展和大城市建设的政策举措，更好统筹教育、民政、社保、卫生等民生领域政策。

　　提升城市管理水平。巩固提升全国文明城市创建成果，把柯桥区、上虞区纳入全国文明城市建设范围。实施智慧城市建设规划，精心编制专项规划，推进重点项目建设。健全城市管理考评体系，探索公建设施市场化运营模式，促进城市管理科学化、标准化、精细化。深入推进“三改一拆”，改造旧住宅区、旧厂区和城中村350万平方米，拆除违法建筑300万平方米以上，进一步做好拆用结合工作。启动市区生活垃圾转运、处置等重大环卫基础设施建设，开展生活垃圾分类收集试点。持续加大交通治堵力度，加快交通“四网”建设，建立市区道路维修统筹协调机制，联合开展“治超治限”，严管严治交通违法行为，建成绍兴交通运行指数平台，积极创建公交优先示范城市。加强古城保护性开发，有序推进部分公共服务向古城外转移。完善社区规划，理顺管理体制，强化队伍建设，提升社区服务能力。规范发展小餐饮、小旅社等“六小行业”，大力整治城市乱象。

　　推进城乡联动发展。开展诸暨省级“美丽县城”建设试点，加快诸暨城东新城、城西产业新城建设。制定实施嵊新区域融合发展规划政策和行动计划，加快重大基础设施、公共服务设施共建共享。实施小城市培育新三年行动计划，规划建设一批聚焦产业、兼具文化内涵和旅游功能的特色小镇。深入开展美丽乡村“四级联创”，做好历史文化村落保护利用，加快推进“空心村”改造建设。大力扶持乡村旅游、农业休闲观光等富农产业，鼓励发展家庭农场，积极壮大村级集体经济，努力促进农民增收。完善农民创业就业服务体系，提高农民培训质量，造就一支适应现代农业发展的高素质职业农民队伍。稳步推进农村产权制度和户籍制度改革，把有意愿进城的农民有序转化为城镇居民。继续加大对山区、老区的帮扶力度，加快欠发达地区发展步伐。着力完善以交通为重点的城乡基础设施，做好城市轨道交通1号线、杭甬高速孙端互通、金甬铁路绍兴段、杭温铁路绍兴段等项目前期工作，开工建设解放路南延、孙曹公路袍江段等工程，加快推进绍诸高速诸暨延伸线、杭金衢高速拓宽等工程建设，建成越兴路南延等项目。继续优质高效推进杭绍台高速各项工作，确保今年控制性节点动工建设。

　　加强城乡环境整治。牢固树立“绿水青山就是金山银山”的理念，花更大的力气保护好“绿水青山”，建设生态文明。深入推进“五水共治”，加快推进“双百双千”工程，继续实施钦寸水库、高湖滞洪区改造、鉴湖水环境整治、长乐江堤防、上虞世纪新丘围涂等工程建设，实现省最严格水资源管理制度考核目标。扎实推进城镇生活污水截污纳管，完成规模畜禽养殖场治理。因地制宜、保质保量开展农村生活污水治理，落实长效运行管理措施。完善饮用水源地和生态功能区生态补偿机制，加大湿地保护力度，创建汤浦水库省级自然保护区。持续开展“五气合治”，完成燃煤锅炉炉外脱硫脱硝改造，建立用能确权、交易等市场化机制，确保市区空气质量优良率达到68%以上。推广工业污泥、建筑泥浆、工程渣土“五统一”制度。广泛开展“四边三化”。加强市花兰花、市树香榧的宣传推广，推动市花、市树进城入户，深入建设“森林绍兴”、“花卉绍兴”，力争创建成为国家森林城市。

　　（四）坚持以人为本，全面提升社会治理水平

　　完善社会保障体系。健全促进就业、鼓励创业、自主择业的体制机制，加强公共就业信息服务平台建设，完善大学生就业创业帮扶政策，力争新增城镇就业10万人，培养各类技能人才5万人。健全企业工资集体协商制度和最低工资标准调整机制，提高企业退休人员基本养老金待遇和城乡居民低保标准，基本实现城乡养老保险、医疗保险全覆盖。健全城乡居民临时救助制度，大力发展红十字、慈善等公益事业，强化困难群体、困境儿童、残疾人救助帮扶，实现全市残疾人意外伤害保险全覆盖。健全养老服务体系，扶持发展养老产业，探索建立“医养结合”养老新模式，争创全国智慧居家养老服务标准化试点。

　　加快社会事业发展。加强公共文化服务体系建设，广泛开展文化惠民活动，扩大政府购买文化服务范围。加大文化精品创作支持力度，办好第27届中国戏剧梅花奖大赛。推进教育优质均衡发展，加强学校内涵建设，继续实施学前教育新三年行动计划，探索中考招生制度改革，落实高考配套改革，全力创建绍兴大学。积极推进职业教育产教融合、校企合作。深化基层卫生综合改革和公立医院改革，稳步探索实施分级诊疗制度，启动市立医院运行筹备，确保通过国家卫生城市复查。办好第八届市运会，申办2017年国际皮划艇马拉松世界杯，争创省体育强市。

　　加强社会治理创新。坚持发展“枫桥经验”，扎实推进“平安绍兴”建设。深化“枫桥式”镇街、民主法治村（社区）创建，提高乡村治理水平。注重法治宣传教育质量，切实加强基层司法所建设，积极构建公共法律服务体系，建立健全大调解工作网络。全面实行流动人口“积分制”管理，促进流动人口优质化。完善立体化治安防控体系，创新“四化融合、三位一体”安全生产管理体制，加强食品药品和农产品质量安全监管，保障人民群众生命健康安全。提升气象服务水平，加强应急能力建设，健全防灾救灾体系。推进社会组织健康有序发展，充分发挥社会组织功能和作用。健全社会诚信体系，打造“信用绍兴”。加强虚拟社会管理，倡导互联网文明。做好国防建设、国防教育、国防动员、人民防空和双拥优抚、国家安全等工作。发展民族宗教、外事侨务、对台事务等事业，支持工会、共青团、妇联开展工作。

　　（五）坚持依法行政，全面推进法治政府建设

　　依法全面履行职责。完善法治政府建设考核评价体系，推行政府集体学法制度，深化政府法律顾问制度。坚持法定职责必须为、法无授权不可为，开展法外设权、规外收费等清理整顿，建立健全行政裁量权基准制度。加大简政放权力度，推进各级政府事权规范化，严格行政规范性文件制定程序。深化行政执法体制改革，探索乡镇综合执法，建立行政执法和刑事司法衔接机制，坚持严格规范公正文明执法。健全行政机关依法应诉制度，提高行政复议透明度。坚持问需于民、问计于民，提高决策科学化、民主化水平。精心编制“十三五”规划，科学引领经济社会发展。

　　强化行政权力监督。自觉接受人大依法监督、政协民主监督，加强政府内部层级监督、审计监督和行政监察、政府督查，主动接受社会监督、舆论监督。认真贯彻新预算法，建立财政专项资金绩效第三方评价制度。优化公共资源配置，促进公共资源交易信息化、便利化、规范化。深入推进政务公开，进一步加强财政预算决算、重大建设项目实施、社会公益事业建设等领域的政府信息公开，健全民生实事、有效投资、财政专项资金等向社会通报制度，让权力在阳光下运行。

　　深入开展作风建设。巩固和拓展党的群众路线教育实践活动成果，开展“三严三实”专项教育，持续加大“四风”整治力度，高标准贯彻中央八项规定和省、市有关精神。深化领导干部社会化评价，强化行政问责，大力整治庸懒散奢浮粗、“为官不为”，切实增强干部责任心、执行力和担当精神。深入贯彻厉行节约、反对浪费各项规定，严把公务消费关，把更多财力用在急需的民生事业上。健全廉政风险防控机制，严明政治纪律、组织纪律、工作纪律，严惩腐败行为，树立廉洁高效的政府形象。

　　各位代表，每年办好一批民生实事，是我市坚持多年的一项重要制度。今年，我们继续向社会公开征集，明确了十个方面的民生实事：（1）淘汰黄标车1.2万辆、燃煤锅炉500台以上，切实减少废气排放，让人民群众看到更多的蓝天白云。（2）新增农村生活污水治理受益农户12万户，完成河道整治145公里、清淤250万立方米，全面消除黑臭河、垃圾河，让水乡的水变得更绿、更清。（3）新增公共自行车服务网点180个、投放公共自行车5000辆以上，让人民群众出行更方便、更健康。（4）建成保障性安居住房5600套、分配6300套，让更多的人民群众实现住有所居。（5）建成绍兴平水江水厂，加快推进宁绍特高压输变电工程，开展天然气滨海新城门站及高压管道工程前期工作，让我们的城市水、电、气保障更完善、更安全。（6）启用绍兴市“社会保障市民卡”，实现诊疗、公交、旅游等“一卡通”；有效推进智慧居家养老工程建设，为越城区范围内有需求的70周岁以上城市老年人免费配置“一键通”，让我们的社会保障更加信息化、人性化。（7）全面整治规范小作坊、小餐饮和小食杂店，完成农贸市场改造20家以上；实施义务教育学校校园直饮水工程，全市覆盖率达70%以上，让人民群众吃得更放心、更安心。（8）引导和帮助城镇失业人员再就业3.5万人，让更多的人民群众可以通过劳动创造自己的美好生活。（9）全面实现“绍兴人免费游绍兴”，让全市人民免费畅游市里的景点。（10）建成400个以上“E邮柜”等电子商务投递终端，启用移动智能医疗服务系统，让人民群众更好地享受现代化、智能化、个性化的服务。对这十方面工作，市政府将进一步加大财政投入，健全工作机制，及时公布进展情况，努力把民生实事办实办好。

　　各位代表！新的一年我们任务艰巨、责任重大、使命光荣。让我们更加紧密地团结在以习近平同志为核心的党中央周围，在中共绍兴市委的坚强领导下，紧紧依靠全市人民，同心同德、同向同行，干在实处、走在前列，为建设现代化绍兴、创造更加美好的明天而努力奋斗！
